# Impact of post-decannulation high fever on mortality in patients with severe ARDS treated with veno-venous ECMO: a multicenter retrospective study

**DOI:** 10.1186/s40560-026-00865-8

**Published:** 2026-02-11

**Authors:** Kenji Fujizuka, Mitsuaki Nishikimi, Kazuya Kikutani, Ryo Emoto, Shinichiro Ohshimo, Shigeyuki Matsui, Nobuaki Shime, Hiroyuki Suzuki, J-CARVE registry group, Junki Ishii, Jun Hamaguchi, Kazuki Matsumura, Keiki Shimizu, Mitsunobu Nakamura, Mamoru Masuda, Yoshihiro Hagiwara, Takayuki Ogura, Ryuichi Nakayama, Naofumi Bunya, Junichi Maruyama, Yosuke Matsumura, Yoshitaka Ogata, Yu Amemiya, Masayuki Yagi, Yutaro Furukawa, Hayato Taniguchi, Noriyuki Hattori, Shinichi Kai, Tokuji Ikeda

**Affiliations:** 1 Department of Critical Care and Emergency Medicine, Red Cross Maebashi Hospital, Maebashi, Japan; 2 Department of Emergency and Critical Care Medicine, Graduate School of Biomedical and Health Sciences, Hiroshima University, 1-2-3 Kasumi, Minami-Ku, Hiroshima, 734-8551 Japan; 3 Department of Emergency and Critical Care Medicine, Aichi Medical University, Nagakute, Japan; 4 Department of Biostatistics, Nagoya University Graduate School of Medicine, Nagoya, Japan

**Keywords:** Respiratory distress syndrome, Body temperature, Hospital mortality, Infection

## Abstract

**Background:**

Few studies have examined the prognostic impact of high fever after decannulation from veno-venous (V-V) extracorporeal membrane oxygenation (ECMO) in patients with severe acute respiratory distress syndrome (ARDS). We aimed to investigate the incidence and prognostic significance of post-decannulation high fever in this population, exploring its association with mortality, stratified by the presence of infectious complications at decannulation.

**Methods:**

This study was a post hoc analysis of a multicenter retrospective registry that included adult patients with severe ARDS who were successfully weaned off V-V ECMO between 2012 and 2022 across 24 institutions in Japan. High fever was defined as a core body temperature of ≥ 39.0 °C within 3 days after ECMO decannulation. The primary outcome was 90-day in-hospital mortality. Cox proportional hazards models were used to examine the association between post-decannulation high fever and mortality. As a subgroup analysis, we evaluated this association according to the presence or absence of infectious complications.

**Results:**

Among 522 patients, 121 (23.2%) developed high fever within 3 days after ECMO decannulation. In the overall cohort, 90-day in-hospital mortality did not differ significantly between the high-fever and no-fever groups (19.0% vs. 13.7%, *p* = 0.372). Multivariable analysis showed no statistically significant association between high fever and mortality (hazard ratio [HR] 0.92, 95% confidence interval [CI] 0.55–1.56, *p* = 0.770). Subgroup analyses revealed opposite associations depending on infection status. High fever was associated with reduced mortality in patients with infection (HR 0.33, 95% CI 0.12–0.89, *p* = 0.045) but increased mortality in those without (HR 2.25, 95% CI 1.23–4.11, *p* = 0.011).

**Conclusions:**

Post-decannulation high fever occurs in nearly one-fourth of patients with severe ARDS treated with V-V ECMO. Its association with mortality appears to differ depending on the infection status at decannulation, underscoring the importance of carefully assessing infectious complications.

## Background

Veno-venous (V-V) extracorporeal membrane oxygenation (ECMO) is a life-support technology used to provide extracorporeal gas exchange for patients with severe acute respiratory distress syndrome (ARDS) [1]. Decannulation from V-V ECMO is attempted once the patient’s native lung function is deemed sufficiently recovered [2]. However, this process can occasionally be complicated by the deterioration of circulatory, respiratory, or hemostatic status post-decannulation [3].

Post-decannulation high fever is one of the most frequently observed complications after V-V ECMO decannulation [4] and can further destabilize patients’ hemodynamic and respiratory status. This occurs because elevated body temperature increases oxygen consumption, which is accompanied by elevated respiratory and heart rates [5–7]. Additionally, ECMO support can induce systemic inflammatory response syndrome (SIRS); moreover, fever may be masked while receiving ECMO support, only to manifest shortly after ECMO decannulation [8].

Despite the importance of understanding the epidemiology and impact of post-decannulation fever on patient outcomes, only a few studies have addressed this topic. Existing studies are limited to single-center analyses, often including patients receiving veno-arterial (V-A) ECMO as well as those receiving V-V ECMO, and there are no large-scale multicenter studies focusing specifically on V-V ECMO [3, 8]. Importantly, previous large ICU cohort studies in non-ECMO populations have demonstrated that the prognostic impact of body temperature differs according to infection status at ICU admission [9, 10]. However, whether this relationship applies to patients treated with V–V ECMO, who experience profound immune and inflammatory alterations that can make interpretation of fever particularly complex, remains unknown. Moreover, fever after ECMO decannulation may arise from mechanisms that are not fully captured in other settings (e.g., thrombosis or inflammatory responses related to the cannulation/decannulation process), underscoring the clinical importance of evaluating the temperature–mortality association specifically in ECMO patients.

The aim of this study was to describe the occurrence of post-decannulation high fever in patients with severe ARDS and to investigate its effect on mortality using a retrospective database of patients with severe ARDS requiring V-V ECMO from 24 institutions across Japan [11]. We also aimed to evaluate the relationship between high fever and mortality, stratified by the presence or absence of infectious complications.

## Methods

### Study design

This study was conducted as a post hoc analysis of the J-CARVE registry, a retrospective database of patients with severe ARDS receiving V-V ECMO, which includes chest computed tomography (CT) data obtained at the start of ECMO. The J-CARVE registry was registered in the University Hospital Medical Information Network Clinical Trials Registry prior to data collection (UMIN000048709). Details of the registry are provided in our previous report [9]. In brief, the registry includes data on patients’ basic demographics and comorbidities, laboratory data, mechanical ventilation (MV) settings and measured values, treatments, outcomes, and anonymized chest CT data. Information on maximum core body temperature, presence of complicated infections, and infection foci on days 1, 2, and 3 after ECMO decannulation was also retrospectively collected.

This study was approved by the Institutional Review Board of Japan Red Cross Maebashi Hospital (202,262). The board waived the need to obtain informed consent from patients to ensure participant anonymity, as stipulated by Japanese government guidelines.

### Participants

The J-CARVE registry contains data on adults (aged ≥ 18 years) with severe ARDS who received V-V ECMO support between January 2012 and December 2022 at any of the 24 participating intensive care units (ICUs). Severe ARDS was diagnosed based on the Berlin criteria (ratio of arterial oxygen partial pressure to fractional inspired oxygen [P/F ratio] ≤ 100) [12]. Patients who did not achieve V-V ECMO decannulation, who died within 3 days after V-V ECMO decannulation because the exposure of interest (maximum body temperature within the first 3 days after decannulation) could not be fully assessed, or who had missing body temperature data on at least one of the first 3 days after decannulation were excluded from this study.

### V-V ECMO management

The participating hospitals adhered to guidelines for the indication, management, and decannulation of V-V ECMO [13, 14]. ECMO support was considered for patients with hypoxemic respiratory failure, defined as a P/F ratio of < 150 mmHg on high fractional inspired oxygen (> 0.9) with optimized positive end-expiratory pressure. During ECMO support, MV settings were adjusted to achieve lung-protective ventilation. Oxygenation targets were set at an arterial oxygen partial pressure of 55–65 mmHg, and tidal volume was reduced to maintain a plateau pressure below 30 cm H_2_O. Core body temperature was maintained between 36.0 and 38.0 °C using the ECMO heat exchanger.

Once lung function improved, extracorporeal blood flow rates were gradually reduced to 2.0 L/min, gas flow was tapered, and the system was turned off for 2–12 h. If arterial blood gas and respiratory parameters remained stable, the ECMO system was removed.

### Definition of post-decannulation fever

Patients were categorized as having high fever if their core body temperature reached ≥ 39.0 °C at least once within 3 days after ECMO decannulation. This threshold was selected to capture physiologically significant hyperthermia that may cause vital sign instability. The definition was based on previous observational studies [15] and supported by a systematic review discussing prognostic implications of fever intensity in critically ill patients [16]. The core body temperature was measured via the ECMO circuit during support (when available) and via urinary catheter after decannulation.

### Definition of infectious complication after ECMO decannulation

Infection status was defined retrospectively based on whether infectious complications requiring antimicrobial therapy occurred within 3 days after ECMO decannulation. Infectious complications were clinically diagnosed by the treating physicians during the hospital stay, using a comprehensive assessment of microbiological culture results, imaging findings, and clinical symptoms, in accordance with the Centers for Disease Control and Prevention National Healthcare Safety Network surveillance definitions [17]. Patients without such a diagnosis were classified as “without infection”, and positive cultures without compatible clinical signs of infection were considered colonization. This definition included infections that were diagnosed retrospectively during the hospital stay. For example, a patient may undergo a fever workup on day 2 after ECMO decannulation and initially be assessed as having “fever without infection”. If the culture obtained on day 2 subsequently becomes positive on day 4 and the treating physicians revise the diagnosis to “fever with infection”, initiating antimicrobial therapy for an infection deemed to have been present on day 2, the patient was classified as having an infectious complication within 3 days after ECMO decannulation.

### Timing of post-decannulation clinical variables

Arterial blood gas (ABG) parameters were defined as the first available measurements obtained after ECMO decannulation, within a maximum of 6 h. Ventilator settings were defined as the most frequently used settings within the first 24 h after ECMO decannulation. The use of vasopressors was defined as any administration within 24 h after ECMO decannulation.

### Outcomes

The primary outcome was 90-day in-hospital mortality, defined as mortality occurring within 90 days of ECMO initiation.

### Statistical analyses

The Mann–Whitney U test and Chi-square test were used to compare continuous and categorical variables, respectively, among baseline characteristics. To investigate the association between high fever within 3 days after V-V ECMO decannulation and 90-day in-hospital mortality, we conducted Kaplan–Meier survival analyses using the log-rank test and multivariable Cox proportional hazards regression analyses. The survival time from V-V ECMO initiation was considered uncensored if the patient died in the hospital on or before day 90 (with day 0 being the day of ECMO decannulation). Survival times were censored on the day of discharge from the hospital or on day 90.

For the multivariable Cox regression, we constructed two models using Firth’s correction to account for the relatively large number of covariates. The primary model (Model 1) included the presence of infectious complications at ECMO decannulation and five clinical variables selected a priori based on previous reports [18, 19]: age, the Murray lung injury score at ECMO initiation, use of neuromuscular blockers during ECMO support, use of corticosteroids during ECMO, and the P/F ratio at ECMO decannulation. The secondary model (Model 2) utilized a reduced set of covariates, comprising age, the P/F ratio at ECMO decannulation, and vasopressor use at ECMO decannulation.

To address missing data in these variables, we employed multiple imputation by chained equations using the “mice” package [20], and pooled estimates were obtained from 20 imputed datasets according to Rubin’s rules.

As a subgroup analysis, we conducted Kaplan–Meier survival analysis and multivariable Cox proportional hazards regression analysis with the same adjustment factors (model 1 and 2), stratified by the presence or absence of infectious complications at ECMO decannulation. Interaction effects between the presence of infection and spiking fever on mortality were evaluated using an adjusted Cox proportional hazards regression model (model 1).

We performed a sensitivity analysis restricted to patients with objectively confirmed infectious complications (pneumonia, urinary tract infection [UTI], or bacteremia) to address the concern that the infection definition used in our main analysis might be subjective or unclear. Pneumonia was defined by a Clinical Pulmonary Infection Score (CPIS) ≥ 6 [21], UTI by bacterial colony counts ≥ 10^5^ CFU/L on urine culture [22], and bacteremia by positive blood culture results.

In addition, to assess how the hazard ratio (HR) for 90-day in-hospital mortality was influenced by the maximum body temperature within 3 days after ECMO decannulation, spline curves were estimated using the same adjustment variables with model 1. Variability in the estimated curves was assessed using the bootstrap method (10,000 times).

All reported p-values were two-sided, and statistical significance was set at p < 0.05. All analyses were performed using R version 4.1.1 (The R Foundation for Statistical Computing, Vienna, Austria). The “mice” package [20] was used for multiple imputation, and the “mgcv” package [23] was used for spline curve estimation in R.

## Results

**Fig. 1:**
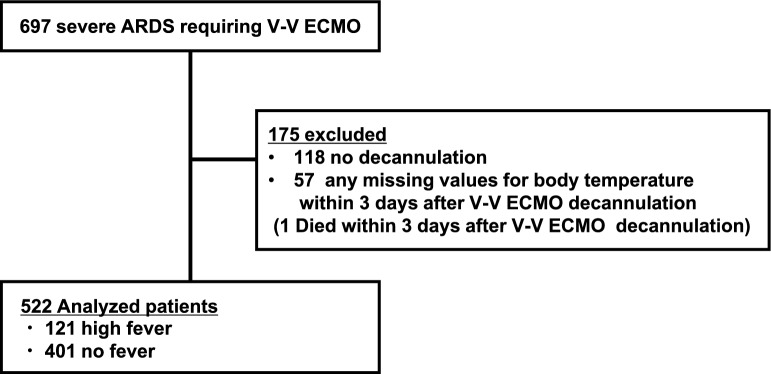
Patient flow. Abbreviations: ICU, intensive care unit; ARDS, acute respiratory distress syndrome; V-V ECMO, veno-venous extracorporeal membrane oxygenation

Among 697 patients with severe ARDS requiring V-V ECMO at 24 participating ICUs in Japan, 175 were excluded (Fig. 1). Among the 522 patients whose data were analyzed, 121 (23.2%) experienced high fever (≥ 39ºC) within 3 days after ECMO decannulation, whereas 401 (76.8%) did not.

The baseline characteristics of the patients are summarized in Table 1. Patients in the high-fever group were younger (54.0 [44.5–65.5] vs. 59.0 [49.0–66.0] years) and exhibited higher rates of neuromuscular blocker use (67.5% [81/121] vs. 55.6% [223/401]) but lower rates of steroid use (55.8% [67/121] vs. 70.2% [280/401]) during ECMO. No significant differences were observed in the primary etiology of ARDS, the Murray lung injury score at ECMO support initiation, or ECMO duration. Use of vasopressors, the P/F ratio, partial pressure of carbon dioxide, and MV settings at ECMO decannulation also did not differ significantly between the two groups.

**Table 1:** Baseline characteristics of all patients

	High fever (+) (*n* = 121)	High fever (−) (*n* = 401)	*P*
Age, y	54.0 (44.5–65.5)	59.0 (49.0–66.0)	0.009
Sex, male, *n* (%)	91 (75.2)	312 (77.8)	0.553
BMI, kg/m^2,a^	26.1 (22.9–30.2)	26.2 (23.0–30.5)	0.779
Past medical history			
Chronic kidney disease, *n* (%)	6 (5.0)	36 (9.0)	0.134
COLD, *n* (%)	10 (8.3)	54 (13.4)	0.112
Interval from the start of MV to ECMO initiation, days^b^	2.0 (1.0–4.0)	2.0 (1.0–4.0)	0.420
Primary etiology of ARDS, *n* (%)			0.673
Bacterial pneumonia	20 (16.5)	58 (14.5)	
Viral pneumonia	64 (52.9)	236 (58.9)	
Other pneumonia	29 (24.0)	80 (20.0)	
Extra-pulmonary	8 (6.6)	27 (6.7)	
Murray lung injury score^c^	3.25 (3.00–3.50)	3.25 (2.75–3.50)	0.090
Use of neuromuscular blockers during ECMO, *n* (%)^d^	81 (67.5)	223 (55.6)	0.019
Steroid use during ECMO, *n* (%)	67 (55.8)	280 (70.2)	0.003
ECMO duration, days^e^	10 (7–16)	9 (7–15)	0.193
At ECMO decannulation			
Use of vasopressor, *n* (%)^f^	47 (39.2)	149 (37.4)	0.732
P/F ratio^g^	213 (168–278)	230 (188–289)	0.071
PaCO_2_, mmHg^h^	43 (39–48)	44 (40–50)	0.449
PEEP values, cmH_2_O^i^	10 (8–12)	10 (8–12)	0.361
Dynamic driving pressure, cmH_2_O^j^	11 (7–12)	12 (8–12)	0.185

Data are presented as the medians and interquartile ranges (25th–75th percentile), or as absolute frequencies with percentages

Abbreviations: *BMI* body mass index; *COLD* chronic obstructive lung disease; *MV* mechanical ventilation; *ECMO* extracorporeal membrane oxygenation; *ARDS* acute respiratory distress syndrome; *P/F ratio* PaO_2_/F_I_O_2_ ratio; *PaCO₂* partial pressure of arterial carbon dioxide; PEEP, positive end-expiratory pressure

^a^Missing value = 1

^b^Missing value = 1

^c^Missing value = 24

^d^Missing value = 1

^e^Missing value = 1

^f^Missing value = 4

^g^Missing value = 14

^h^Missing value = 5

^i^Missing value = 15

^j^Missing value = 57

^g^Missing value = 5

After excluding 5 patients with missing data, infectious complications were observed in 39.7% (27/120) of patients with high fever and 20.7% (41/397) of those without high fever, respectively (p < 0.001). Among the 68 patients clinically diagnosed with infection, 30 (44.1%) had a positive blood culture testing. Regarding the site of infection, 33 patients (48.5%) had lung infection, 1 (1.4%) had urinary tract infection, 21 (30.9%) had device-related infection, and 4 (5.9%) had sepsis with an unknown focus. Although pulmonary and device-related infections were prevalent, no significant difference was found between the groups (p = 0.319). The baseline characteristics of the patients with and without infectious complications are summarized in Tables 2 and 3, respectively. Among patients with infectious complications, there were no significant differences in baseline characteristics between the high-fever and no-fever groups (Table 2). In contrast, among patients without infectious complications, those in the high-fever group were significantly younger and had higher rates of neuromuscular blocker use but lower rates of steroid use during ECMO compared with those in the no-fever group (Table 3).

**Table 2:** Baseline characteristics of the patients with infection at extracorporeal membrane oxygenation decannulation

	High fever (+) (*n* = 27)	High fever (−) (*n* = 41)	P
Age, y	62.0 (50.0–70.0)	62.0 (53.5–67.0)	0.960
Sex, male, *n* (%)	22 (81.5)	32 (78.1)	0.731
BMI, kg/m^2^	25.9 (23.3–29.9)	25.4 (22.4–29.0)	0.344
Past medical history			
Chronic kidney disease, *n* (%)	6 (5.0)	36 (9.0)	0.134
COLD, *n* (%)	1 (3.7)	3 (7.3)	0.534
Interval from the start of MV to ECMO initiation, days	3.0 (1.0–4.0)	2.0 (1.0–4.0)	0.833
Primary etiology of ARDS, *n* (%)			0.737
Bacterial pneumonia	4 (14.8)	6 (14.6)	
Viral pneumonia	15 (55.6)	27 (65.9)	
Other pneumonia	5 (18.5)	6 (14.6)	
Extra-pulmonary	3 (11.1)	2 (4.9)	
Murray lung injury score^a^	3.25 (2.75–3.50)	3.25 (3.00–3.25)	0.444
Use of neuromuscular blockers during ECMO, *n* (%)	16 (59.3)	27 (65.9)	0.582
Steroid during ECMO, *n* (%)	17 (63.0)	33 (80.5)	0.112
ECMO duration, days	10 (8–18)	11 (8–19)	0.895
At ECMO decannulation			
Use of vasopressor, *n* (%)^b^	15 (57.7)	16 (39.0)	0.135
P/F ratio	198 (145–250)	203 (157–256)	0.950
PaCO2, mmHg	42 (39–47)	44 (40–51)	0.379
PEEP values, cmH2O^c^	10 (8–12)	10 (8–12)	0.740
Dynamic driving pressure, cmH2O^d^	12 (9.5–13.3)	11 (4.3–13.5)	0.144
Positive blood culture	11 (40.7)	19 (46.3)	0.649
Infection site			0.319
Lung	12 (44.4)	21 (51.2)	
Device	11 (40.7)	10 (24.4)	
Others	4 (14.8)	10 (24.4)	

Data are presented as the medians and interquartile ranges (25th–75th percentile), or as absolute frequencies with percentages

Abbreviations: *BMI* body mass index; *COLD* chronic obstructive lung disease; *MV* mechanical ventilation; *ECMO* extracorporeal membrane oxygenation; *ARDS* acute respiratory distress syndrome; *P/F ratio* PaO2/FIO2 ratio; *PaCO₂* partial pressure of arterial carbon dioxide; PEEP, positive end-expiratory pressure

^a^Missing value = 5

^b^Missing value = 1

^c^Missing value = 2

^d^Missing value = 6

**Table 3:** Baseline characteristics of the patients without infection at extracorporeal membrane oxygenation decannulation

	High fever (+) (*n* = 94)	High fever (−) (*n* = 360)	*P*
Age, y	52.0 (42.8–63.0)	58.5 (49.0–66.0)	0.001
Sex, male, *n* (%)	69 (73.4)	280 (77.8)	0.371
BMI, kg/m^2,a^	26.4 (22.6–30.5)	26.4 (23.1–30.7)	0.562
Past medical history			
Chronic kidney disease, *n* (%)	5 (5.3)	33 (9.2)	0.208
COLD, *n* (%)	9 (9.6)	51 (14.2)	0.226
Interval from the start of MV to ECMO initiation, days^b^	2.0 (1.0–4.3)	2.0 (1.0–4.0)	0.597
Primary etiology of ARDS, *n* (%)			0.588
Bacterial pneumonia	16 (17.0)	52 (14.4)	
Viral pneumonia	49 (52.1)	209 (58.1)	
Other pneumonia	24 (25.5)	74 (20.6)	
Extra-pulmonary	5 (5.3)	25 (6.9)	
Murray lung injury score^c^	3.25 (3.00–3.50)	3.25 (2.75–3.50)	0.109
Use of neuromuscular blockers during ECMO, *n* (%)^d^	65 (69.9)	196 (54.4)	0.006
Steroid during ECMO, *n* (%)	50 (53.8)	247 (69.0)	0.007
ECMO duration, days^e^	10 (7–15)	9 (7–14)	0.266
At ECMO decannulation			
Use of vasopressor, *n* (%)^f^	32 (34.0)	133 (37.3)	0.564
P/F ratio^g^	226 (178–281)	233 (192–291)	0.148
PaCO_2_, mmHg^h^	44 (39–49)	44 (40–50)	0.761
PEEP values, cmH_2_O^i^	10 (8–12)	10 (8–12)	0.263
Dynamic driving pressure, cmH_2_O^j^	11 (8–12)	11 (7–12)	0.463

Data are presented as the medians and interquartile ranges (25th–75th percentile), or as absolute frequencies with percentages

Abbreviations: *BMI* body mass index; *COLD* chronic obstructive lung disease; *MV* mechanical ventilation; *ECMO* extracorporeal membrane oxygenation; *ARDS* acute respiratory distress syndrome; *P/F ratio* PaO2/FIO2 ratio; *PaCO₂* partial pressure of arterial carbon dioxide; PEEP, positive end-expiratory pressure

^a^Missing value = 1

^b^Missing value = 1

^c^Missing value = 19

^d^Missing value = 1

^e^Missing value = 1

^f^Missing value = 3

^g^Missing value = 14

^h^Missing value = 5

^i^Missing value = 13

^j^Missing value = 51

**Fig. 2:**
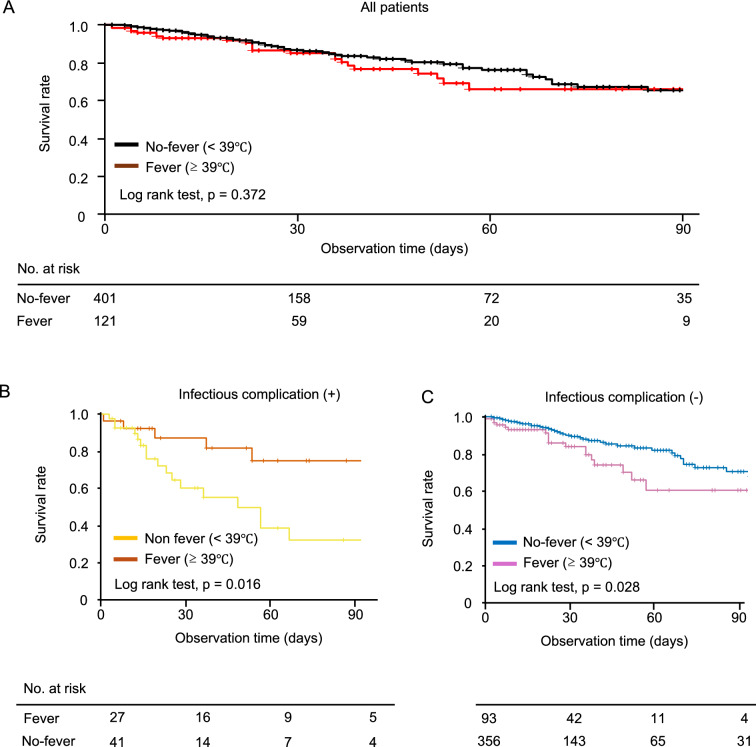
Survival curves for patients with and without fever after extracorporeal membrane oxygenation decannulation. The 90-day in-hospital mortality rates were compared between the patients with and without high fever (**A**). We also evaluated the mortality separately according to the presence (**B**) or absence (**C**) of infectious complications. Five patients were excluded owing to missing values for infectious complications

The 90-day in-hospital mortality was 19.0% (23/121) in the high-fever group and 13.7% (55/401) in the no-fever group, with no significant difference between them. Kaplan–Meier curves were plotted for the patients in the high-fever and no-fever groups, respectively (p = 0.372, Fig. 2A). In the adjusted Cox regression analyses, no statistical difference was observed for the association between high fever and their mortality (HR 0.92, 95% confidence interval [CI] 0.55–1.56, *p* = 0.770, Table 4).

**Table 4:** In-hospital mortality within 90 days of ECMO initiation in all analyzed patients

	Model 1		Model 2	
Variable	Adjusted HR (95% CI)	*P*	Adjusted HR (95% CI)	*P*
High fever	0.92 (0.55, 1.56)	0.770	0.94 (0.56, 1.59)	0.830
Infectious complication at ECMO decannulation	1.88 (1.09, 3.24)	0.027	1.89 (1.10, 3.25)	0.024
Age	1.01 (0.99, 1.03)	0.365	1.01 (0.99, 1.03)	0.285
Murray lung injury score	1.02 (0.91, 1.13)	0.738		
Use of neuromuscular blockers during ECMO	1.08 (0.68, 1.72)	0.755		
Use of corticosteroids during ECMO	1.23 (0.74, 2.06)	0.423		
P/F ratio at ECMO decannulation	0.49 (0.26, 0.93)	0.032	0.49 (0.27, 0.90)	0.026
Use of vasopressor at ECMO decannulation			1.83 (1.17, 2.87)	0.011

Multivariate analyses were performed using six adjusting factors (Model 1: presence of infectious complications at ECMO decannulation, age, the Murray lung injury score at ECMO initiation, use of neuromuscular blockers during ECMO support, use of corticosteroids during ECMO, and the P/F ratio at ECMO decannulation) or four adjusting factors (Model 2: presence of infectious complications at ECMO decannulation, age, the P/F ratio at ECMO decannulation, and vasopressor use at ECMO decannulation)

Abbreviations: *ECMO* extracorporeal membrane oxygenation; *P/F ratio* PaO2/FIO2 ratio; *HR* hazard ratio; *CI* confidence interval

As a subgroup analysis, we then investigated the association between high fever and mortality, stratified by the presence or absence of infectious complications at ECMO decannulation. Distinct trends emerged when mortality was analyzed separately based on the presence or absence of infectious complications. The mortality rates were 18.5% (5/27) and 41.5% (17/41) in the high-fever and no-fever groups, respectively, in the presence of infection, whereas these were 19.4% (18/93) and 10.7% (38/356), respectively, in the absence of infection. The log-rank tests showed that the mortality was greater in the no-fever group than in the high-fever group among the patients with infectious complications (*p* = 0.016, Fig. 2B), whereas the mortality was greater in the high-fever group than in the no-fever group among the patients without infectious complications (*p* = 0.028, Fig. 2C). There was a significant interaction effect of high fever and infectious complications on mortality (*p* < 0.001). The adjusted Cox regression analyses showed that high fever was associated with decreased mortality in patients with infection (HR 0.33, 95% confidence interval [CI] 0.12–0.89, *p* = 0.045, Table 5), whereas it was associated with increased mortality in patients without infection (HR 2.25, 95% CI 1.23–4.12, *p* = 0.011, Table 6). Sensitivity analyses restricted to patients with objectively confirmed infections (pneumonia with CPIS ≥ 6, urinary tract infection with ≥ 10^5^ CFU/L on urine culture, or bacteremia with positive blood culture) showed results consistent with those of the primary analysis (Table 7).

**Table 5:** Subgroup analysis: in-hospital mortality within 90 days of ECMO initiation in patients with infection

	Model 1		Model 2	
Variable	Adjusted HR (95% CI)	*P*	Adjusted HR (95% CI)	*P*
High fever	0.33 (0.12, 0.89)	0.045	0.30 (0.11, 0.80)	0.028
Age	1.00 (0.96, 1.05)	0.913	1.01 (0.97, 1.06)	0.533
Murray lung injury score	1.01 (0.84, 1.22)	0.897		
Use of neuromuscular blockers during ECMO	0.73 (0.27, 1.94)	0.537		
Use of corticosteroids during ECMO	0.84 (0.29, 2.41)	0.753		
P/F ratio at ECMO decannulation	0.87 (0.23, 3.32)	0.839	1.08 (0.31, 3.81)	0.906
Use of vasopressor at ECMO decannulation			2.23 (0.89, 5.57)	0.104

Multivariate analyses were performed using five adjusting factors (Model 1: age, the Murray lung injury score at ECMO initiation, use of neuromuscular blockers during ECMO support, use of corticosteroids during ECMO, and the P/F ratio at ECMO decannulation) or three adjusting factors (Model 2: age, the P/F ratio at ECMO decannulation, and vasopressor use at ECMO decannulation)

Abbreviations: *ECMO* extracorporeal membrane oxygenation; *P/F ratio* PaO2/FIO2 ratio; *HR* hazard ratio; *CI* confidence interval

**Table 6:** Subgroup analysis: in-hospital mortality within 90 days of ECMO initiation in patients without infection

	Model 1		Model 2	
Variable	Adjusted HR (95% CI)	*P*	Adjusted HR (95% CI)	*P*
High fever	2.25 (1.23, 4.11)	0.011	2.19 (1.23, 3.89)	0.010
Age	1.01 (0.99, 1.04)	0.186	1.01 (0.99, 1.03)	0.219
Murray lung injury score	1.01 (0.88, 1.17)	0.861		
Use of neuromuscular blockers during ECMO	1.10 (0.64, 1.90)	0.733		
Use of corticosteroids during ECMO	1.45 (0.80, 2.61)	0.222		
P/F ratio at ECMO decannulation	0.38 (0.18, 0.79)	0.013	0.37 (0.18, 0.75)	0.008
Use of vasopressor at ECMO decannulation			1.95 (1.15, 3.31)	0.016

Multivariate analyses were performed using five adjusting factors (Model 1: age, the Murray lung injury score at ECMO initiation, use of neuromuscular blockers during ECMO support, use of corticosteroids during ECMO, and the P/F ratio at ECMO decannulation) or three adjusting factors (Model 2: age, the P/F ratio at ECMO decannulation, and vasopressor use at ECMO decannulation)

Abbreviations: *ECMO* extracorporeal membrane oxygenation; *P/F ratio* PaO2/FIO2 ratio; *HR* hazard ratio; *CI* confidence interval

**Table 7:** Sensitivity analyses for multivariate Cox regression analysis of 90 days in-hospital mortality from ECMO decannulation

	Infectious complication (+)		Infectious complication (−)	
Variable	Adjusted HR (95% CI)	*P*	Adjusted HR (95% CI)	*P*
High fever	0.26 (0.08, 0.85)	0.045	1.93 (1.07, 3.48)	0.033
Age	1.01 (0.96, 1.07)	0.583	1.01 (0.99, 1.04)	0.179
Murray lung injury score	1.16 (0.92, 1.45)	0.231	0.99 (0.86, 1.13)	0.862
Use of neuromuscular blockers during ECMO	0.93 (0.32, 2.70)	0.891	1.05 (0.61, 1.80)	0.868
Use of corticosteroids during ECMO	0.29 (0.09, 0.92)	0.058	1.45 (0.81, 2.61)	0.221
P/F ratio at ECMO decannulation	0.91 (0.22, 3.81)	0.902	0.39 (0.19, 0.83)	0.018

Abbreviations: *ECMO* extracorporeal membrane oxygenation; *P/F ratio* PaO2/FIO2 ratio; *HR* hazard ratio; *CI* confidence interval

**Fig. 3:**
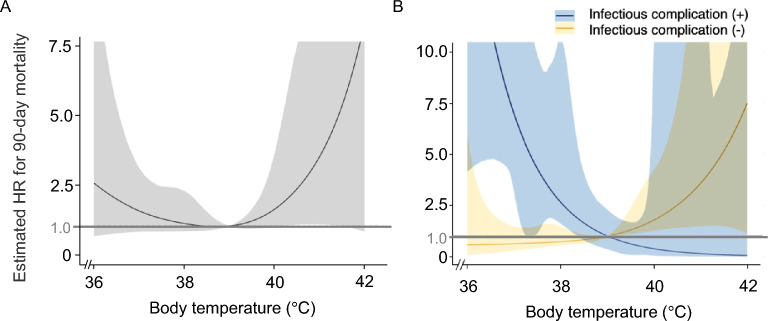
Spline curves showing the association between maximum body temperature and mortality risk after ECMO decannulation. The spline curve for all analyzed patients (**A**) and two subgroup spline curves for patients with (blue line in **B**) and without (yellow line in **B**) infectious complications were plotted. *HR* hazard ratio; *ECMO* extracorporeal membrane oxygenation

Spline curves for all patients and for those with and without infectious complications were plotted to evaluate how the HR for 90-day mortality varied with the maximum body temperature within 3 days after ECMO decannulation (Fig. 3). In the overall cohort, the curve showed an inverted U-shaped pattern, without statistically significant differences across most temperature levels. In contrast, the subgroup curves demonstrated opposite trends, indicating that lower body temperature was associated with a higher HR for mortality among patients with infectious complications, whereas higher body temperature was associated with a higher HR for mortality among patients without infectious complications.

## Discussion

High fever following ECMO decannulation is one of the most common complications of V-V ECMO. Despite its clinical relevance, few studies have investigated this phenomenon. This is the first multicenter study on the incidence and mortality of high fever after ECMO decannulation in patients with severe ARDS who underwent V-V ECMO. In our study, approximately 20% of patients developed high fever within 3 days of ECMO decannulation, and approximately 40% of these patients had infectious complications at ECMO decannulation.

The incidence of high fever after ECMO decannulation in our cohort was lower than those reported in previous single-center studies [3, 8]; this discrepancy may be attributed to differences in the patient populations studied. Previous studies have included patients receiving V-A and V-V ECMO, regardless of underlying conditions. Patients receiving V-A ECMO have higher prevalence rates of pneumonia and bloodstream infections [24]. Additionally, V-A ECMO delivers oxygenated blood directly into the arterial system, which may result in thrombi and inflammatory cytokines from the ECMO circuit being directly transported to the peripheral organs, potentially causing SIRS [25]. The novelty of our study lies in its exclusive focus on patients undergoing V-V ECMO.

Fever is widely recognized as a protective physiological reaction to infection [26]. Previous reports showed that an increase in peak body temperature during the first 24 h after ICU admission in critically ill patients with infection being associated with reduced mortality [9] or with no increase in mortality [10]. Our findings extend these observations to post-decannulation fever in patients with severe ARDS treated with V–V ECMO, demonstrating that high fever after ECMO decannulation is associated with lower mortality among those with infectious complications.

In contrast, in our study, fever after ECMO decannulation was associated with increased mortality in patients without infection. A few previous studies in non-ECMO populations have suggested that elevated body temperature is associated with increased mortality in the absence of infection, and that this association may be modified by the presence or absence of infectious complications [9, 10]. Our findings are consistent with these reports, although the mechanisms underlying noninfectious fever after ECMO decannulation may differ from those of other clinical settings.

Several potential causes of noninfectious fever after ECMO decannulation have been proposed, including thrombosis [27]. Although thrombosis could not be evaluated in our study, previous reports suggest that thrombotic events after ECMO decannulation are relatively common [28]. Another potential cause of post-decannulation fever is the persistence of SIRS. Although respiratory function may have improved sufficiently to allow for ECMO withdrawal, incomplete control of the underlying disease and/or exposure of blood to the ECMO circuit may contribute to sustained systemic inflammation [29, 30], and temperature control with an external heat exchanger may mask fever while on ECMO [31]. Therefore, fever after decannulation may represent, at least in part, persistent systemic inflammation. Overall, noninfectious fever and its prognostic implications are likely multifactorial, and it should be interpreted as a physiological reflection of the patient’s underlying clinical status, rather than a direct causative factor for mortality.

Our findings underscore the importance of carefully assessing infectious complications at the time of ECMO decannulation when interpreting the prognostic significance of fever. However, given the retrospective and observational nature of this study, the observed associations should not be interpreted as evidence for specific therapeutic interventions, such as decisions regarding antibiotic use or routine screening for thrombosis. Further prospective and interventional studies are needed to establish evidence-based strategies for managing post-decannulation fever in both infectious and noninfectious contexts and to determine whether tailored clinical responses can improve patient outcomes.

This study has some limitations. First, as this was a retrospective, registry-based study conducted in Japan, we could not obtain several factors that could significantly influence the results. For example, data such as post-decannulation steroid and antibiotic information, Acute Physiology and Chronic Health Evaluation II scores, Sequential Organ Failure Assessment scores, and detailed infection severity were unavailable. In addition, detailed causes of death were not systematically collected in the registry. Second, despite sensitivity analyses restricting the cohort to patients with objectively confirmed infectious complications, we cannot completely exclude the possibility that some patients classified as “without infection” may have had occult or subsequently diagnosed infections. Third, we could not investigate the potential causal relationship between high fever and conditions such as systemic inflammatory response syndrome or thrombosis. Fourth, the impact of hypothermia on mortality was not analyzed because no patients had a peak body temperature ≤ 36.0 °C within 3 days after ECMO decannulation. Finally, we excluded nearly 10% of the eligible cohort owing to missing temperature data. But, the results of our sensitivity analyses using imputed body temperature values were similar to those of the main analyses (unpublished data).

## Conclusions

Among patients with severe ARDS treated with V-V ECMO, 23.2% developed high fever within 3 days after ECMO decannulation. Post-decannulation high fever was associated with decreased mortality in patients with infectious complications at the time of ECMO decannulation, whereas it was associated with increased mortality in patients without infectious complications. These findings indicate that the prognostic significance of post-decannulation fever differs according to infection status and underscore the importance of carefully evaluating infectious complications at ECMO decannulation. Further prospective studies are warranted to confirm these observations.

## Data Availability

The datasets used and analyzed in the current study are available from the corresponding author upon reasonable request.

## Abbreviations

ICU: Intensive care unit
ARDS: Acute respiratory distress syndrome
V-V: Veno-venous
V-A: Veno-arterial
ECMO: Extracorporeal membrane oxygenation
CT: Computed tomography
SIRS: Systemic inflammatory response syndrome
MV: Mechanical ventilation
HR: Hazard ratio
CI: Confidence interval
UTI: Urinary tract infection
CPIS: Clinical Pulmonary Infection Score
